# Structural pattern matching of nonribosomal peptides

**DOI:** 10.1186/1472-6807-9-15

**Published:** 2009-03-18

**Authors:** Ségolène Caboche, Maude Pupin, Valérie Leclère, Phillipe Jacques, Gregory Kucherov

**Affiliations:** 1 Computer Science Laboratory of Lille, UMR USTL/CNRS 8022, INRIA, F59655, Villeneuve d'Ascq, France; 2 ProBioGEM (UPRES EA 1026), University of Sciences and Technologies of Lille, F59655, Villeneuve d'Ascq, France

## Abstract

**Background:**

Nonribosomal peptides (NRPs), bioactive secondary metabolites produced by many microorganisms, show a broad range of important biological activities (e.g. antibiotics, immunosuppressants, antitumor agents). NRPs are mainly composed of amino acids but their primary structure is not always linear and can contain cycles or branchings. Furthermore, there are several hundred different monomers that can be incorporated into NRPs. The NORINE database, the first resource entirely dedicated to NRPs, currently stores more than 700 NRPs annotated with their monomeric peptide structure encoded by undirected labeled graphs. This opens a way to a systematic analysis of structural patterns occurring in NRPs. Such studies can investigate the functional role of some monomeric chains, or analyse NRPs that have been computationally predicted from the synthetase protein sequence. A basic operation in such analyses is the search for a given structural pattern in the database.

**Results:**

We developed an efficient method that allows for a quick search for a structural pattern in the NORINE database. The method identifies all peptides containing a pattern substructure of a given size. This amounts to solving a variant of the maximum common subgraph problem on pattern and peptide graphs, which is done by computing cliques in an appropriate compatibility graph.

**Conclusion:**

The method has been incorporated into the NORINE database, available at . Less than one second is needed to search for a pattern in the entire database.

## Background

Nonribosomal Peptides (NRPs) are bioactive compounds having various important biological functions (e.g. as antibiotics, siderophores, antitumor agents, immunosuppressants). NRPs are synthesized by large multi-enzymatic complexes called Nonribosomal Peptide Synthetases (NRPSs) that are modularly organized [1]. Each module is responsible for the incorporation of a specific monomer and is itself subdivided into domains catalysing specific enzymatic reactions.

Until about fifteen years ago, the number of known NRPs remained relatively low. However, many new molecules have been reported in the literature during the last years, associated with different biological activities and having a broad range of potential applications. This triggered a considerable interest among the research community in the nonribosomal synthesis pathway.

Among potential applications of such studies, redesigning natural products by genetic engineering of NRPSs opens an interesting new way in drug discovery [2]. Indeed, modifying the nucleotide sequence of a natural NRPS or combining modules of different NRPSs could potentially yield a more efficient compound or a product with a new biological activity. However, generating a new peptide with a specific function from a modified NRPS nucleic sequence requires a deep understanding of both the assembly line and the resulting products.

NRPS enzymes have been well studied for several years. Stachelhaus *et al*. [3] discovered a specificity-conferring code of adenylation domains. With this discovery, several software programs have been developed [4-6] to predict a produced peptide from the NRPS protein sequence. With the increasing number of sequenced genomes, the number of hypothetical NRPSs increases too. Therefore, this raises the problem of verifying whether a peptide predicted to be produced by a NRPS is already known or even corresponds to a part of a known peptide.

NRP molecules show several important particularities. The first one is related to the incorporation of non-proteinogenic amino acids. Indeed, in addition to the twenty standard amino acids found in proteins, several hundreds of other residues can be encountered in final NRPS products. Incorporated residues can further undergo chemical modifications such as epimerisation or methylation. Products of other biosynthesis pathways, like lipids or carbohydrates, can also be introduced. Because of this composition diversity of NRPs, we will use the term 'monomer' rather than 'amino acid' for NRP structural units. Another interesting property of NRPs is their structure. Unlike regular proteins, the primary structure of NRPs is not always linear but can also be cyclic (partially or totally), branched or even poly-cyclic. A computational treatment of these molecules appears therefore to be very different from standard proteins and requires a development of specific computational methods and resources.

There exist, however, very few computational resources specifically devoted to NRPs and, until recently, there was no one providing a complete inventory of those. To fill this lack, we have developed the NORINE database [7] which is the first resource entirely dedicated to NRPs. It contains various annotations of each peptide such as the producing organism, bibliographic references, activities and, most importantly, its monomeric structure. The choice of representing NRP molecules by their monomeric rather than atomic structure reflects the way they are synthesized by successive addition of monomers. This structure is encoded by an undirected labeled graph representing its (possibly nonlinear) structure. Using undirected (rather than directed) edges is justified by the existence of nonpeptide bonds, appearing e.g. in cyclic or branched peptides, for which the orientation can not be naturally defined. Furthermore, using directed edges could be restrictive for the analysis of peptide families: for example, lipopeptides containing an asparagine-serine dipeptide include the iturin family (produced by different *Bacillus *species). Tsuge *et al*. [8] proposed a model in which iturin or mycosubtilin swapped nucleotide sequences encoding adenylation domains after a common ancestor became established. In this case, looking for a directed asparagine-serine dipeptide would miss mycosubtilin that has a serine-asparagine dipeptide.

Similar to the search for sequence patterns in genomic and protein databases, NORINE raises the need to efficiently search for *structural *patterns. In the simplest case, one needs to identify if a given peptide is already present in the database. An even more important motivation is provided by the close relation between the structure and the function of the peptide. For example, Minowa *et al*. [9] identified motifs that are significantly related to some biological activities. Therefore, a search for a structural pattern can help to assign a biological function to a peptide under study.

In some analyses, one needs to identify a part of the pattern, rather than the whole pattern, occurring in a given peptide. For example, the order of monomers in the resulting peptide can be changed with respect to the order of modules in the synthetase (so-called nonlinear biosynthesis [10]). For instance, in the biosynthesis of syringomycin [11], the *Syr*B1 gene responsible for the incorporation of the threonine monomer is located upstream of the *Syr*E gene in the genome, whereas threonine is the final monomer of the peptide. Therefore, a search for the entire pattern predicted from the synthetase does not produce an output, while a search for a common substructure allows one to identify the peptide.

In this paper, we present an efficient method to identify a substructure of a given structural pattern that occurs in a given NRP, where both the pattern and the peptide are represented by undirected labeled graphs. From the computational viewpoint, this can be expressed as a variant of the Maximum Common Subgraph (MCS) problem, which is NP-complete [12] i.e. is very unlikely to be solvable by an algorithm with a running time polynomially bounded on the graph size. Another related NP-complete problem, called Graph Motif problem [13], is to look for a connected subgraph with the given (multi-)set of labels. Despite of the formal NP-completeness of the underlying computation, our method works very efficiently on NRP graphs, taking advantage of their relatively small size and specific structural properties.

Our method is based on the commonly used construction of a Compatibility Graph (CG), also called association or product graph, in which the largest clique represents a solution to the MCS problem (see [14] for a review). Here we adapt the method of CG to the structural search for nonribosomal peptides. We propose several ways to reduce the size of the CG, both in terms of number of nodes and edges. Note that the size of the CG is a crucial factor for the efficiency of the whole method, as the clique search in the CG is the computationally most demanding step. We follow the idea of filtration by trying to detect, as early as possible, pairs of nodes that cannot be mapped one to the other by a graph morphism. This considerably reduces the size of the CG and leads to an efficient practical structural search for nonribosomal peptides. The presented algorithm has been implemented in NORINE. Here we present some experimental results showing the efficiency of the method. We also provide some examples of using structural search for nonribosomal peptides in biological studies.

## Results and discussion

### Theory and algorithms

#### Graph representation of NRP structure

**Figure 1:**
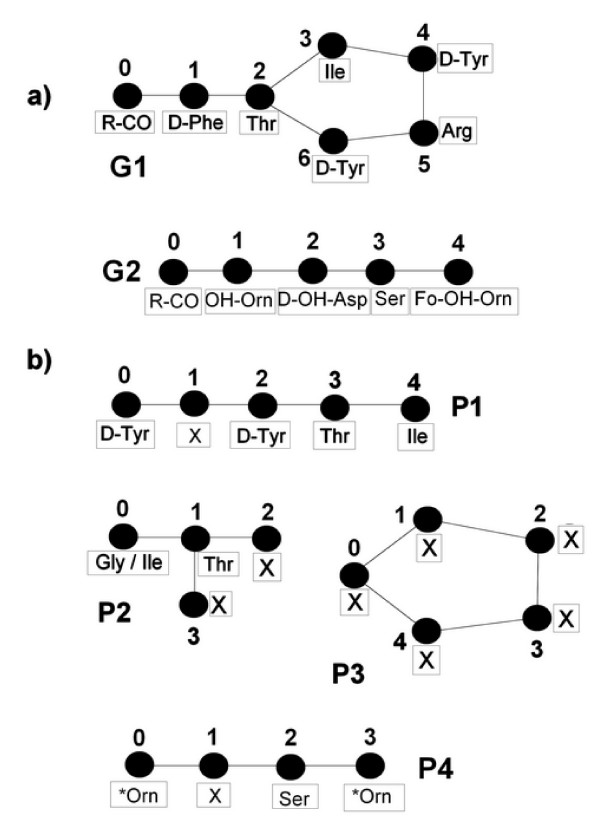
**Examples of peptide and pattern graphs**. This figure shows examples of (a) peptide graphs and (b) pattern graphs. Nodes and edges represent monomers and chemical bonds respectively. Labels are the monomer names.

We encode the monomeric structure of NRPs by an undirected labeled graph. A *peptide graph *is a graph *G *= (*V*, *E*, *M*, *f*), where *V *is a set of nodes, *E *⊆ *V *× *V *is a set of undirected edges i.e. pairs (*u*, *v*) with *u*, *v *in *V*, and *f*: *V *→ *M *a labeling mapping of nodes. Here nodes represent monomers, edges correspond to chemical bonds between monomers and labels are monomer names. Monomer names are encoded by a set of simple rules inspired by the IUPAC nomenclature [15]: monomers are denoted by the classical three-letter code, possibly preceded by a symbol of a chemical modification. For example, *NMe-Ala *represents the N-methyl-alanine monomer. Each node in a graph has a unique number in order to distinguish two nodes with the same label. Figure 1(a) shows examples of peptide graphs.

A structural pattern is also represented by a graph. Let *P *= (*V*_*P*_, *E*_*P*_, *L*, *f*) be a pattern graph, where *V*_*P *_is a set of nodes, *E*_*P *_⊆ *V*_*P *_× *V*_*P *_a set of undirected edges and *f*: *V*_*P *_→ *L *is the labeling of nodes. The main difference between graphs *G *and *P *is in the set of possible labels: *M *⊂ *L *but *L *contains some additional labels. One of them is the "joker label", denoted 'X', that stands for any monomer. *L *also includes alternative labels denoted by lists of several monomers separated by the '/' symbol. The intended meaning is that any monomer of the list can occur at the corresponding position. Finally, *L *also includes labels formed by the '*' symbol followed by a monomer. This means that any derivative of the monomer can be found at this position. Figure 1(b) shows some examples of structural patterns. For example, in pattern P4, *Orn means that at this position, ornithine (Orn) or any of its derivatives, such OH-Orn or Fo-OH-Orn, can be found.

#### Computing a maximal common substructure using the compatibility graph

The construction of the compatibility graph (CG) is often used in chemoinformatics to establish a structure mapping between two molecule graphs [14]. The CG encodes potential mappings between the two graphs. Then, a search for the largest clique in the CG allows one to obtain the maximum common subgraph. First, we describe the classical CG construction.

#### Compatibility graph

The classical definition of the CG of two graphs *P *and *G *is as follows:

• the set of nodes of CG is the cartesian product *V*_*P *_× *V*, i.e. a node *U *(*u*, *u'*) of CG corresponds to the association of a pattern node *u *and a peptide node *u'*; in the case of (unambigously) labeled nodes, only nodes with the same label get associated to form a node of the CG,

• nodes *U *(*u*, *u'*) and *V *(*v*, *v'*) are adjacent in the CG if and only if *u *≠ *v *and *u' *≠ *v' *and one of the following conditions holds:

(1) - *u *is adjacent to *v *in *P *and *u' *is adjacent to *v' *in *G*

(2) - *u *is not adjacent to *v *in *P *and *u' *is not adjacent to *v' *in *G*

For our purposes, we modify the classical CG definition to only require that associated nodes have compatible labels. If *f *(*u*) ∈ *M *(i.e. the label of *u *is not 'X' nor a "*-label" monomer), then any peptide node *u' *with *f *(*u'*) = *f *(*u*) gets associated with *u*. If *f *(*u*) = 'X', then *u *gets associated with any node *u' *of *G*. Finally, if *f *(*u*) is a "*-label", then *u *naturally gets associated with any node *u' *labeled by a derivative of the corresponding monomer.

**Figure 2:**
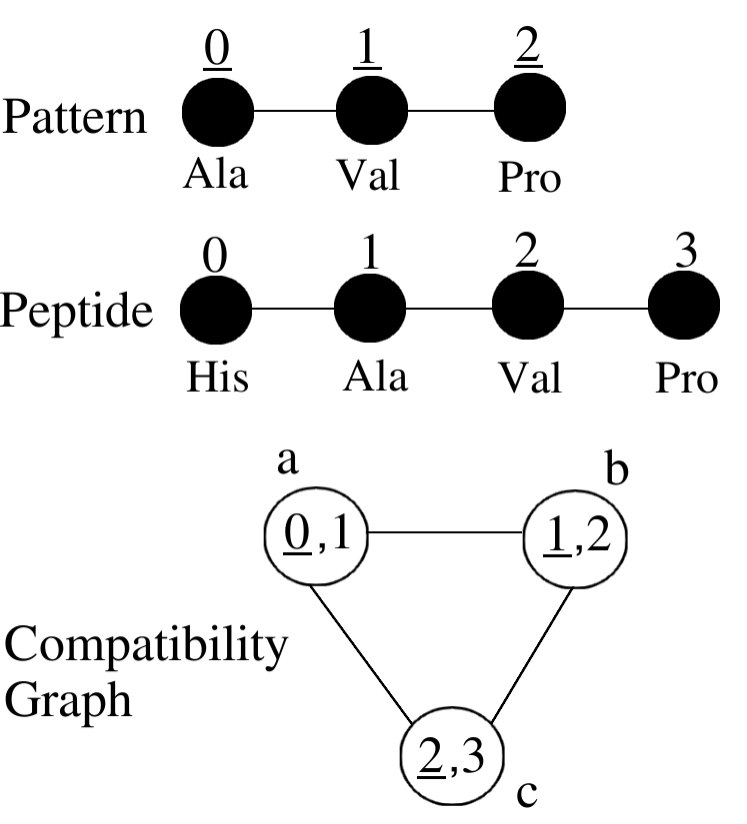
**A simple example of compatibility graph**. The Figure shows a pattern graph, a peptide graph and the corresponding CG. Each node of pattern and peptide graphs has a label (for example 'Ala') and a number that is a unique identifier of this node. Identifiers of pattern nodes are underlined in order to distinguish them from peptide nodes. A node of the CG corresponds to the association of a node of the pattern graph (underlined number) and a node of the peptide graph with the same label. Nodes of the CG are named by letters. For example, node 'a' corresponds to the association of pattern node 0 and peptide node 1 that have both label 'Ala'. Edges between nodes 'a' and 'b' and between nodes 'b' and 'c' in the CG correspond to condition (1). Edge between nodes 'a' and 'c' corresponds to condition (2) of the definition of CG. A clique of size 3 exists in the CG and corresponds to the occurrence of the pattern in the peptide.

Figure 2 shows a simple example of CG of a pattern and a peptide graph. Edges between nodes 'a' and 'b' and nodes 'b' and 'c' correspond to condition (1). The edge between nodes 'a' and 'c' corresponds to condition (2).

#### Clique computation

The CG represents all potential mappings between graphs *P *and *G*. Recall that a clique in an undirected graph is a subset of nodes such that every two nodes of this subset are connected by an edge. Each clique in the CG corresponds to a common substructure of graphs *P *and *G*, whose size (number of nodes) is equal to that of the corresponding clique. Consequently, searching for a clique of a given size *k *(*k*-clique) is equivalent to searching for a common subgraph of size *k*. In Figure 2, nodes a, b and c form a 3-clique, which corresponds to the occurrence of the whole pattern in the peptide. The general clique detection problem i.e. finding whether there is a *k*-clique in a graph is NP-complete [12].

#### Refining CG building rules

Our goal is to detect efficiently and exactly whether a part (connected subgraph) of a size *k *of a pattern graph *P *is a substructure of a peptide graph *G*. We assume that parameter *k *is specified by the user. If *k *is equal to the size of the pattern graph, the problem amounts to checking if *P *is a substructure of *G*. In other words, the searched pattern *P *occurs in the tested peptide *G*. The notion of "substructure" needs to be made precise. In the above construction of CG, a clique corresponds to a common *induced *subgraph of both *P *and *G *(see [14]). In our case, we want to allow a node of *G *to have more incident edges than the associated node of *P*. For example, we want pattern P1 from Figure 1 to match the peptide graph G1, although there is no edge between the first and the last node in P1 while there is one between the corresponding nodes in G1. In mathematical terms, we are looking for a subset of *k *nodes in *P *such that the corresponding induced subgraph of *P *is connected and occurs as a (not necessarily induced) subgraph of *G*. This asymmetry between *P *and *G *prevents using standard solutions for computing common substructures (see [14]) and raises the need to develop an efficient method appropriate for our setting. For this purpose, we modify the above solution based on clique search in the compatibility graph.

We first modify the definition of compatibility graph, taking into account that if two nodes in *G *are connected by an edge, the associated nodes in *P *may or may not be connected. Since the size of the CG (both in terms of the number of nodes and edges) is the crucial factor for efficiency, we need to make sure to keep this size reduced and filter out those node associations which cannot participate in the mapping. Even prior to constructing the CG, we verify simple properties that prevent a common substructure of size *k *to exist. First, the size of *G *must be greater than or equal to *k*. Furthermore, at least *k *nodes of the pattern must be associated to some nodes of the peptide graph. Only if these two simple tests are verified, we proceed to the construction of the CG and searching for a *k*-clique.

#### CG nodes

In order to decrease the number of nodes in the CG, we associate a node *u' *of *G *and a node *u *of *P *only if the degree of *u' *is greater than or equal to the degree of *u*. This is justified by the above definition of common substructure of *P *in *G*.

#### CG edges

According to our definition of common substructure, we have to modify the above definition of an edge in the CG. Conditions (1) and (2) are replaced by the following:

(3) • nodes *U *(*u*, *u'*) and *V *(*v*, *v'*) are adjacent in the CG if and only if *u *≠ *v *and *u' *≠ *v' *and *u' *is adjacent to *v' *in *G provided that u *is adjacent to *v *in *P*

In other words, if two nodes in the pattern graph are connected, then the corresponding nodes in the peptide graph must be connected too, but the opposite is not necessarily true. With this definition we achieve that if two nodes *u *and *v *are not connected in the pattern graph *P*, their corresponding nodes *u' *and *v' *in the peptide graph *G *may or may not be connected, in both cases the corresponding nodes *U *(*u*, *u'*) and *V *(*v*, *v'*) in the CG are connected. However, this rule leads to an increase of the number of edges in the CG. Indeed, according to condition (3), the CG has an egde between *U *(*u*, *u'*) and *V *(*v*, *v'*) even if *u *and *v *are not connected in *P *while *u' *and *v' *are connected in *G*. The classical CG constructed according to conditions (1) and (2) would not include an edge in this case. We then introduce a stronger rule in order to reduce the number of edges and to make the search for a *k*-clique efficient. The rule is based on the computation of elementary paths.

An elementary path (EP) in a graph is a path without loops. For each node in *P *and *G*, we compute the size of all EPs from this node to all the others. Since we are interested in connected subgraphs of size *k*, the EP size in such subgraphs is limited to *k *- 1 (which is the maximal number of nodes that can be visited along an EP in a graph of size *k*). For a graph *G*, we store the EP sizes in a matrix *EPS*_*G*_, where the *EPS*_*G *_[*i*, *j*] contains the list of all EP sizes between the nodes *i *and *j*.

**Figure 3:**
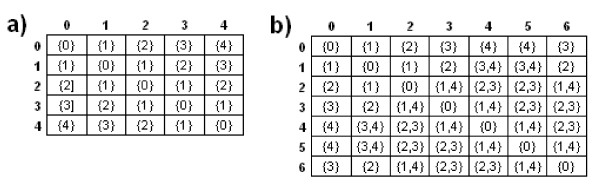
**Matrix of elementary path sizes**. This figure shows matrix of elementary path sizes for (a) P1 of Figure 1 and (b) G1 of Figure 1.

Figure 3 shows the matrices for pattern graph P1 and peptide graph G1 from the Figure 1 with *k *equal to the pattern size. For example, there are two EPs between nodes 1 and 4 in G1, one of size 3 (path 1 - 2 - 3 - 4) and another of size 4 (path 1 - 2 - 6 - 5 - 4). Nodes 0 and 4 are connected by two EPs of size 4 and 5, but the second one is not considered as it is greater than *k *- 1 (P1 has 5 nodes).

We then define an edge between *U *(*u*, *u'*) and *V *(*v*, *v'*) in the CG if and only if the EP size list of (*u*, *v*) in *P *(considered as a multiset) is included in the EP size list of (*u'*, *v'*) in G. This means that the distances between two nodes in P must be included in the respective distances in G in order for an edge in the CG to exist. In other words, the monomers of the EPs beteeen *u *and *v *in *P *and between *u' *and *v' *in *G *are not directly compared but the distances of possible paths in *P *must be also distances of possible paths in *G*. This new rule decreases the number of edges in the CG without losing any information on a possible occurrence of the pattern.

**Figure 4:**
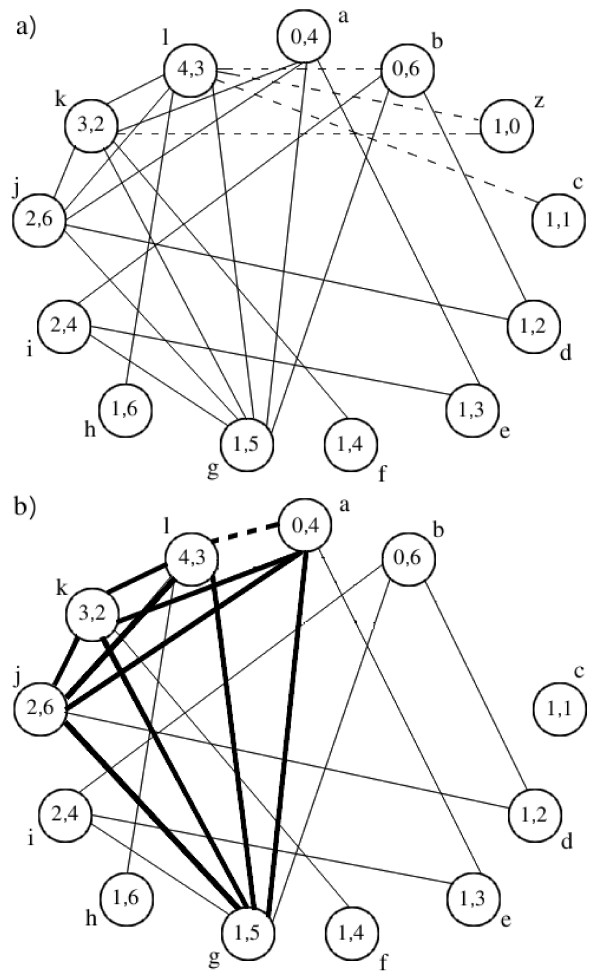
**Example of compatibility graph constructed with classical and new methods**. The CG of pattern graph *P*1 and peptide graph *G*1 of Figure 1 constructed with (a) classical and (b) new CG building rules. Each CG node is identified by a letter. It represents an association between a node of *P*1 and a node of *G*1 with compatible labels. For example, node 'a' associates node 0 of *P*1 and node 4 of *G*1 that both carry the 'D-Tyr' label. Dashed edges correspond to the edges that differ between the two CGs and the bold edges correspond to a clique of size 5 (size of *P*1).

Figure 4 shows the resulting CG of P1 and G1 built with the classical and the new CG building rules, with *k *= 5 (size of pattern P1). Observe that there is no edge between nodes *a *and *l *in the CG constructed with classical building rules (nodes 0 and 4 are not adjacent in *P *whereas nodes 4 and 3 are adjacent in *G*) whereas this edge exists in the CG constructed with the new building rules and implies a clique of size 5 that shows that P1 occurs in G1. In addition, the number of nodes and edges in the two CGs are different: the CG obtained with the classical building rules has 13 nodes and 22 edges whereas the CG obtained with the new building rules has 12 nodes and 19 edges. For example, in the CG obtained with the classical building rules there is an edge between nodes *b*(0, 6) and *l*(4, 3) that does not exist in the CG obtained with the new building rules. This is because the EP sizes between nodes 0 and 4 in P1 (here {4}) are not included in the EP sizes between nodes 6 and 3 in G1 (here {2, 3}). The new CG building rules exclude this kind of edges and thus decrease the overall number of edges in the CG.

#### New CG building rules: summary

We conclude this section by summarizing the CG building rules for a pattern graph *P *and a peptide graph *G*:

• each CG node *U *(*u*, *u'*) corresponds to the association of a node *u *of *P *and a node *u' *of *G *such that *deg*(*u*) ≤ *deg*(*u'*) and *f *(*u*) is compatible with *f *(*u'*).  (4)

• two nodes *U *(*u*, *u'*) and *V *(*v*, *v'*) are adjacent in the CG if and only if *u *≠ *v *and *u' *≠ *v' *and *EPS*_*P *_[*u*, *v*] ⊆ *EPS*_*G *_[*u'*, *v'*]. (5)

#### Search for a *k*-clique

The presence of a *k*-clique in the CG implies that there is an induced subgraph of *P *that is a subgraph of *G*. In the case when *k *is smaller than the size of *P*, we have to verify, in addition, that the corresponding subgraph is connected in *P *(and consequently in *G*).

To search for a *k*-clique, we use a branch and bound algorithm (see Chapter 6 in [16]). It is essentially an exhaustive algorithm that explores the depth-search tree of the graph. For a node of depth *h *in the tree, we try to extend the current clique of size *h *with a new node in order to obtain a clique of size *h *+ 1. The tree is pruned by not exploring the branches with the length smaller than *k*. Once a *k*-clique is found, the search terminates and the pattern occurrence is output.

Another heuristic we use to speed up the clique search is based on the fact that once we identified more than (|*V*_*P*_| - *k*) nodes of pattern that do not participate in the clique, the search for a *k*-clique can be stopped. In the case of search for the entire pattern (*k *= |*V*_*P*_|), each pattern node has to contribute to the clique. For example, in Figure 4(b) with *k *= 5 (pattern size), node 0 of the pattern participates in two nodes *a *and *b *of the graph, which implies that one of these two nodes must belong to the clique. If a *k*-clique containing one of these two nodes is not found, the search is stopped. Finally, another speeding heuristics is to start the search with CG nodes that correspond to pattern nodes of maximal degree and have therefore most chances to lead to a fast detection of non-occurrence of the pattern. Applying all these heuristics leads to a practically fast and exact clique search, as confirmed by experimental results provided in the next section.

### Testing

#### Case study of structural properties of NRPs

**Figure 5:**
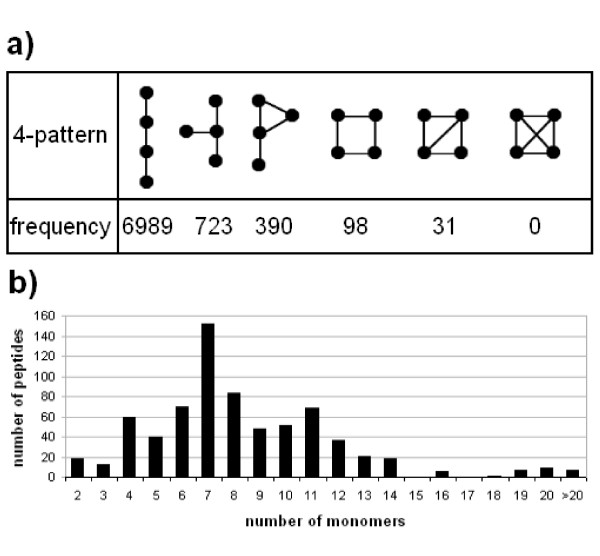
**Structural properties of NRPs contained in NORINE**. Distribution of (a) 4-patterns and (b) peptide sizes in the NORINE database.

We studied the distribution of patterns of size 4 in all peptides of the database. The results are shown in Figure 5(a). They show that the most frequent 4-pattern is the linear pattern. We also computed the distribution of the number of peptide graphs depending on their size. The results are shown in Figure 5(b). More than 70% of peptides have at least seven monomers. This means that a search for a pattern containing seven 'X' labels triggers the construction of the CG for more than 70% of peptides of the NORINE database.

#### Efficiency of the method

In order to test the efficiency of our method, we compared the number of nodes and edges in the CGs obtained with the classical and the new building rules on different examples in the case of search for an entire pattern. The results are shown in Table 1. In the case of matching P3 against G2, the CG constructed with modified rules has no edges because the EP sizes of the pattern are not included in the corresponding EP sizes of the peptide graph. Indeed, P3 is cyclic and each pair of nodes is connected by two EPs whereas G2 is linear and there is only one EP for each pair of nodes. Therefore, our method outputs the answer without looking for a clique. In the last example corresponding to the linear pattern of 19 'X' against alamethicin F50, the difference in the number of nodes (346 against 380) is due to the additional condition on the degree of associated nodes. Moreover, in this example, our version of CG has about 13 times less edges than the CG constructed with the classical rules. These examples illustrate that our method produces a compact compatibility graph, suitable for an efficient clique search.

**Table 1:** Number of nodes and edges of the CG constructed with classical and new building rules

pattern	peptide	# CG nodes	# CG edges
P1	G1	13/**12**	22/**19**
P2	G1	16/**16**	43/**29**
P3	G1	35/**30**	210/**100**
P3	G2	25/**15**	100/**0**
P4	G2	10/**8**	14/**9**
Ala-1^(*a*)^	Ala^(*b*)^	73/**73**	1918/**286**
(X)19^(*c*)^	Ala^(*b*)^	380/**346**	53010/**3948**

Patterns P1–P4 and peptides G1–G2 refer to Figure 1. In all examples, *k *is equal to the pattern size. In columns 3 and 4, data shown in regular and bold font concern respectively the classical and modified CG building rules.

^(*a*) ^linear pattern of size 19 corresponding to alamethicin F50 without the last monomer

^(*b*) ^alamethicin F50 [NORINE:NOR00007]

^(*c*) ^linear pattern of 19 'X' monomers

**Figure 6:**
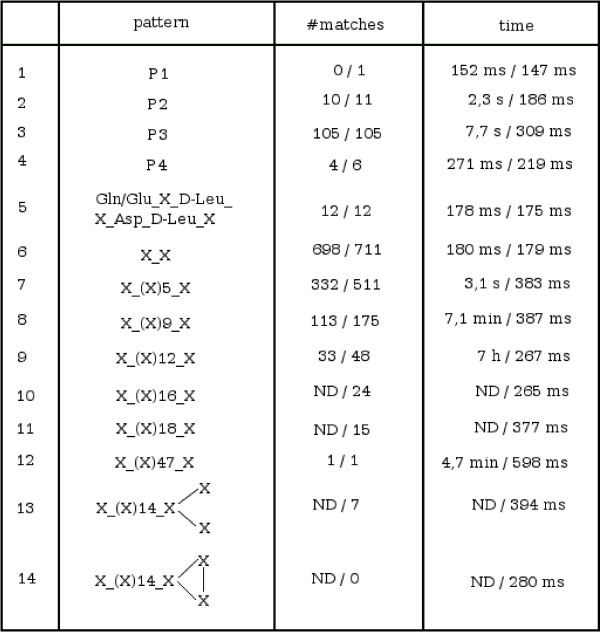
**Search time for different complete patterns in the NORINE database**. Here, *k *is equal to the size of the pattern. In the 2nd and 3rd columns, the first and second value corresponds respectively to the classical and new building rules. 'ND' means that the result has not been obtained as the running time exceeded 8 hours.

In order to validate this speed-up in running time, we measured the search time for different complete patterns in the NORINE database. The results are shown in Figure 6. The first observation is that the number of results is often smaller with the classical rules. This is due to the case when the pattern graph has a number of edges different from the peptide graph. In example 6, we search for peptides containing any pair of monomers which is the case for all 711 peptides of NORINE. However, only 698 peptides are output if the classical building rules are used. There are 13 cyclic dipeptides in Norine. This is due to a special case where two nodes of a peptide are connected by two edges, which corresponds to a cyclisation between the two monomers. This special case cannot be detected with the classical building rules but is taken into account by our method.

For the linear pattern of size 7 (example 7), which is contained in more than 70% of the database peptides, the classical rules show a 8-fold slow-down of the running time compared to the new rules. For a linear pattern composed of 14 'X' (example 9), the classical method required 7 hours to produce the result whereas our method took less than 300 ms. Example 12 is the search for a pattern composed of 49 'X', the size of the largest peptide of the database. About 5 minutes were needed for the classical method to obtain the result whereas our method took only about 600 ms. Example 14 represents a negative test as this pattern does not occur in NORINE. In this example, the classical method did not terminate in 8 hours, whereas our method output the result in 280 ms.

These experiments illustrate the effficiency and adequacy of our method for the search for structural patterns in NRPs.

### Examples of practical applications

In this part, we give some examples of using structural pattern matching of NRPs in biological studies.

#### Structural features

Structural search can allow one to identify a structural motif common to peptides of a given family. As an example, a search for a cyclic 8-node pattern composed of seven 'X' and a fatty acid moiety (represented by the monomer code '*R-'), with *k *= 8, outputs the peptides of the iturin family (iturins, bacillomycins and mycosubtilin), surfactins and lichenysins. Therefore, this pattern represents a common structural feature of this family.

Another example is the search for a pattern associated with a biological activity. For example, pattern P4 occurs in G2 that represents ornibactin. Ornibactin is a siderophore, an iron-chelating molecule. This type of molecule needs bidendate functions that can ensure a six-fold coordination of the ferric iron. Ornithine and its derivatives can harbour this function. A search for complete pattern P4 returns a list of six siderophore peptides such as ornibactin, pyoverdin or foroxymithine. A search for the pattern R-CO_*OH-Orn_*Asp_*Ser_*Orn derived from ornibactin, with *k *= 2, returns a list of 51 peptides. Among them 46 peptides are annotated as siderophores. This example illustrates that structure-function relationships can also be elucidated by searching for a common substructure between a pattern derived from a peptide characterizing the function and the other peptides of the database.

#### Product identification

Another application of structural pattern matching is the search for a predicted peptide. Several studies (see [17,18] for recent examples) start with searching for putative NRPS genes within a genome and predicting the produced peptide out of this sequence. Such a prediction can result in an undetermined monomer or several possible monomers at some positions. The possibility of using 'X' or '/' labels in the structural pattern allows for a look-up for the predicted peptide in the database in order to find out whether the peptide has been discovered before, possibly in another species. To give a concrete example, we submitted six NRPS proteins found in UniProtKB database [19] ([UniProtKB:Q2VQ12–Q2VQ17]) to the NRPSPREDICTOR[6] and obtained predicted peptides. By combining them, we obtained the structural pattern X_NP_X_NP_NP_NP_X_NP_*Leu_X_*Phe/*Trp/*Tyr_*Leu_NP, where NP stands for non-polar amino acids and corresponds to *Val/*Ile/*Leu/*Abu/*Iva in NORINE notation. A search for this complete pattern in NORINE resulted in only one peptide, BT1583, that is consistent with the bibliographic data of UniProtKB. This example illustrates that the structural search can help associating a product with a set of nonribosomal synthetases.

#### Analysis of a putative peptide

From the analysis of protein sequence similarity, some proteins can be predicted as putative NRPSs. Examples of such predictions can be found in the UniProtKB database. Even though the produced peptide has not been identified, one can infer some properties of a putative NRPS product using the structural search. An example can be provided by the putative NRPS [UniProtKB:Q1I964] from UniProtKB found in *Pseudomonas entomophila*. By studying the sequence of this synthetase, four modules can be predicted. Pattern Val_Leu_Ser_Ile is obtained using the NRPSPREDICTOR. This pattern occurs in the lipopeptide putisolvin I stored in NORINE. The search for a more general pattern NP_NP_Ser_NP gives three results, putisolvin I, II and PFL2145, that are all lipopeptides. One can observe that putisolvin I is produced by *Pseudomonas putida*, the same genus of bacteria than [UniProtKB:Q1I694]. This bacteria genus is known to produce various cyclic lipopeptides [20]. By analysing the gene environment of [UniProtKB:Q1I964], we found another gene coding for a putative NRPS [UniProtKB:Q1I963], which probably produces the beginning of the peptide. A condensation domain characteristic of lipopeptide production can be predicted at the begining of the protein. This domain binds the lipid moiety to the peptide part. This is another clue for lipopeptide production. NRPSpredictor outputs the octapeptide X_NP_NP_X_NP_NP_X_Ser which matches no peptide of NORINE. However, the final product would be composed of 12 monomers and a lipid moiety like putisolvin I. In addition, the composition of the predicted peptide does not match any peptide in Norine but is close to the composition of lipopeptides. Indeed, if we compare the monomeric composition of the predicted peptide with putisolvin I, both compositions are similar. Thus, all data converge to a lipopeptide production. This example illustrates that structural pattern search can assist the biological identification of a predicted peptide by inferring its properties.

## Conclusion

Nonribosomal peptides are important bioactive compounds that have various important biological activities and are increasingly studied. With this motivation, we developed the NORINE database [21] that is the first computational resource entirely devoted to NRPs. All peptides stored in NORINE are annotated with their monomeric (possibly non-linear) structure encoded by undirected labeled graphs.

In this paper, we presented an efficient dedicated method to search for a structural pattern in the database. We refined the CG building rules previously used in the literature and improved them to adapt to our problem. The main idea of refinement is to use the information on elementary path sizes and on the node degrees in order to decrease the number of nodes and especially the number of edges in the resulting CG. This, in turn, leads to a considerable speed-up in the search for a clique in the CG, which is the final step in the identification of a pattern occurrence.

As a result, a search for a pattern in the NORINE database currently containing 711 peptide structures takes typically less than one second. Note that the proposed method is exact, i.e. outputs precisely all the peptides that contain the pattern, without any error allowed. Note also that the efficiency of the method can be further increased by pre-computing the matrices of EP sizes for all stored peptides. This would obviously improve the performance of querying the database with different patterns.

Searching for a structural pattern in the database can be used in different biological studies. For example, it can help to identify members of a peptide family that share common structural properties. It can also help to identify a predicted peptide by searching for it in the NORINE database in case the peptide has been discovered before in other species. Furthermore, a search for a structural pattern can provide new insights into peptide features and help to isolate this peptide experimentally. Finally, it can help to elucidate the relationship between structure and function by searching for patterns occurring in peptides that share a common biological activity.

An obvious weakness of the method is that in general it might be unable to identify a common structure if the correspondence is not exact, i.e. some monomers "get replaced" by others (not specified explicitely with the joker or alternative labels), or do not have their counterparts at all. Therefore, an interesting direction for future research would be to extend the method to an "error-tolerant" pattern matching dealing with possible deletions, insertions or substitutions of monomers.

## Methods

### NORINE

The method presented in this paper is included in NORINE. NORINE  is a public Web resource entirely dedicated to NRPs. As of today, NORINE stores 711 peptides, each annotated with different information such as producing organisms, biological activities or its monomeric structure. Monomeric structures are encoded by undirected labeled graphs with nodes representing monomers. Peptides currently stored in NORINE contain overall more than 400 different monomers. Those include all standard amino acids but also many non-standard amino acids incorporated in NRPs during the biosynthesis. Lipids, carbohydrates and polyketides also occur in NRPs and are considered by NORINE as monomers. More details on the NORINE system can be found in [7].

### Implementation

The method has been implemented in Java within the NORINE system. The program looks up for a structural pattern or a common substructure in all the peptides of the NORINE database ([NORINE:NOR00001] to [NORINE:NOR00711] were considered in this publication). All peptides containing the pattern or a common substructure are output. Time measures reported below have been obtained on a PC with a 1.73 GHz processor, 512 MB of RAM and 265 Mflops. The Java code implementing the method can be provided by request to the first author.

## Authors' contributions

SC carried out most of the work on the algorithm design, implementation in NORINE and experiments. MP and GK participated in the algorithmic setup and the design of computational experiments. VL and PJ contributed to various biological issues related to this study. GK and PJ organized the project. SC, MP and GK wrote the manuscript. All authors read and approved the final manuscript.
